# TADM-CGAN: a resting-state to task activation map prediction framework using temporal attention-driven diffusion models and conditional generative adversarial networks

**DOI:** 10.1038/s41598-026-54396-1

**Published:** 2026-05-26

**Authors:** Sasideep Pasumarthi, Nitya Tiwari, Himanshu Padole

**Affiliations:** School of Electrical and Computer Sciences, IIT Bhubaneswar, Khordha, Odisha 752050 India

**Keywords:** Computational biology and bioinformatics, Mathematics and computing, Neuroscience

## Abstract

Predicting task-induced brain activation from resting-state fMRI (rs-fMRI) remains a significant challenge in computational neuroimaging, primarily due to the difficulty in simultaneously modeling the detailed temporal evolution and high spatial resolution of intrinsic neural activity. Most existing literature relies on parcel-based modeling using spatial functional connectivity features, neglecting the long-range temporal interactions and nonlinear fluctuations in rs-fMRI signals. To overcome these limitations, we introduce TADM–CGAN, a two-stage, grayordinate-level cascaded architecture that infers task activation maps directly from rs-fMRI time series, fully utilizing both temporal and spatial characteristics. In the first stage, a multi-head temporal attention–driven diffusion model (TADM) is employed to generate compact temporal embeddings for each grayordinate, capturing dependencies across the entire rs-fMRI time series. These unique temporal features then serve as inputs to 59,412 time series regression models, enabling highly localized, grayordinate-specific prediction of preliminary activation values. In the second stage, a principal component analysis (PCA)-conditioned conditional generative adversarial network (PCA-CGAN) is introduced, where PCA constrains adversarial refinement to a low-rank, biologically meaningful subspace, while the generator with the proposed activation fidelity loss (AFL) reduces noise and sharpens spatial details for the improved predictions. This proposed cascaded framework consistently outperforms prior task activation map prediction methods across a diverse set of task contrasts and datasets, underscoring its robustness and generalizability in practical applications.

## Introduction

Functional magnetic resonance imaging (fMRI), with its many advantages, including high spatial resolution, non-invasive nature, and radiation-free acquisition, has been a widely used neuroimaging modality in recent times^1,2^. By capturing dynamic fluctuations in the blood oxygenation level-dependent (BOLD) signal, fMRI enables examination of both localized and distributed neural processes. Based on their acquisition, the fMRI is broadly categorized into task-based fMRI (t-fMRI) and resting-state fMRI (rs-fMRI). t-fMRI records neural responses during explicit tasks and is typically employed to map functional specialization across cortical and subcortical regions. However, it suffers from limitations such as dependence on subject compliance, variability in task performance, and relatively low test–retest reliability, which constrain its utility in clinical and developmental populations^3,4^. In contrast, rs-fMRI captures spontaneous low-frequency BOLD fluctuations in the absence of task engagement, providing a potential alternative in scenarios where task performance is difficult or unreliable, particularly benefiting non-compliant populations, such as the elderly, infants, or people with disabilities.

To this end, recent studies have explored the feasibility of predicting the t-fMRI activation maps using the rs-fMRI data only. In a seminal work, Tavor et al.^5^ proposed a linear predictive framework for estimating task-induced fMRI activation maps using functional connectivity (FC) maps obtained from rs-fMRI. The preliminary analysis was conducted using group principal component analysis (PCA), followed by group independent component analysis (ICA), which generated 40 spatial FC maps for each hemisphere. Based on the similarity within each hemisphere, 33 cortical FC maps were selected for further analysis. Similarly, for the subcortex region, separate clustering methods were used to generate 32 subcortical features. These FC features, together with a few structural features, resulted in a total of 98 features that were provided to generalized linear regression models (GLM) to obtain the parcel-wise predictions. The evaluation across seven task contrasts in the Human Connectome Project (HCP) dataset^6^, yielded mean correlation values ranging from *r = 0.25* to *r = 0.45*. Following this, Teterva et al.^7^ extended the framework where similar FC maps were used as features in flat and stacked prediction models. However, unlike the simple GLM, they employed sophisticated regressors like elastic net, random forest, XGBoost, and support vector regression (SVR), obtaining a test-retest correlation of 0.7. Cohen et al.^8^also proposed a similar rest-to-task activation prediction framework, which modeled the FC features using non-linear models, viz., bagging and neural networks, resulting in correlations between 0.1 to 0.8. Tik et.al^9^. tested the generalizability of the FC and GLM-based model across datasets and populations by training the model on one dataset and evaluating it on different datasets. The mean correlations obtained for this cross-dataset generalization were between *r = 0.3* and 0.4. Vaibhav et al.^10^ used a different strategy for rest-to-task activation map predictions, mapping the motor contrast in accordance with the pre-surgical planning for the brain tumor. Unlike the aforementioned methods, they modeled using connectome fingerprinting on different parcellations, viz., the multimodal parcellation, the Schaefer parcellation, and a comparison of the Schaefer and multimodal parcellations. A correlation in the range of 0.45 to 0.63 was obtained for all the copes in motor contrasts. Extending to modality-specific predictions, Zhou et al.^11^ demonstrated an accurate prediction of visual cortex activations by introducing Rest2Visual, a volumetric encoder-decoder design that takes 3D features from rs-fMRI as input, modulates them as image embeddings to obtain task activation predictions. This study used a natural scenes dataset, achieving *r = 0.65* between predicted and actual visual task maps.

More recently, with the advent of the field of deep learning, many deep learning-based models have been tried on these predictive frameworks. Ngo et al.^12^ developed a surface-based deep learning framework, brain-surf CNN, that works with a brain’s cortical sheet representation. The resulting predictions, with a dice coefficient between 0.50 and 0.64 and an AUC between 0.20 and 0.30, were on par with the target-repeat reliability of the measured contrast maps. Kwon et al.^13^ used 3D fMRI data as input to SwiFUN, a transformer-based method that used shifted-window attention and contrastive learning to understand complex patterns in sequential data. It demonstrated 27% higher performance in the ABCD dataset compared to the existing methods. Patel et al., in^14^, employed and compared the performance of graph-based, kernel-based, and transformer-based models in the task activation map prediction. The transformer-based models achieved the highest performance, with AUC = 0.82 and correlation values exceeding *r = 0.40*in predicting cognitive scores from rs-fMRI. Sasideep et al.^15^, emphasized on the temporal characteristics of rs-fMRI signal where the windowed histogram-based features obtained from rs-fMRI time series, along with FC features were modeled with a 2D-CNN for the activation map prediction. Their proposed model, GEMReg, yielded the prediction correlations between 0.14 to 0.73 across different task contrasts in the HCP dataset.

However, despite these advances in task activation map predictions, most prediction frameworks still rely on static heuristic FC features that encode spatial dependencies between brain regions, ignoring the temporal dynamics of the rs-fMRI signal, or treat spatial and temporal characteristics independently. Also, their parcel-wise prediction approach assumes a common prediction model for the entire brain parcel, disregarding the subtle variations in grayordinate-specific prediction mappings. To this end, although recent transformer and graph neural networks-based models^13,14^ attempt to exploit the temporal characteristics of rs-fMRI, they operate on parcel-averaged signals or summary connectivity features, thereby discarding the original temporal structure of the BOLD time sequence. Furthermore, transformers introduce substantial computational overhead and require large data sizes to generalize effectively—conditions rarely met in neuroimaging datasets. As a result, existing approaches often struggle to capture the long-range temporal dependencies and fine-grained grayordinate-level dynamics underlying intrinsic brain activity.

To address these limitations, in the present work, we model the input rs-fMRI data as time series data extracted independently from each grayordinate, enabling effective utilization of its rich temporal characteristics. Furthermore, unlike prior works that use a common prediction model for an entire brain parcel, we develop 59,412 separate grayordinate-specific prediction models, enabling fine-grained temporal modeling without spatial averaging. Specifically, our framework introduces TADM-CGAN, a two-stage rs-to-task activation map prediction architecture. In stage 1, a temporal attention–guided diffusion model (TADM) is proposed for an efficient temporal feature extraction, which excels in modeling long-range sequence structure^16,17^ due to its stable likelihood training, noise-based iterative refinement, and multi-scale temporal distribution modeling, potentially outperforming existing transformers, autoencoders, and conventional deep neural networks. It differs from conventional sequence modeling approaches by integrating temporal attention with diffusion-based representation learning. While transformer-based models capture long-range dependencies, they are often sensitive to noise in fMRI signals. In contrast, the diffusion component explicitly models noise corruption and denoising, enabling the extraction of robust latent features. Furthermore, unlike standard diffusion models typically applied in spatial domains (e.g., images), the proposed approach adapts diffusion to the temporal domain of rs-fMRI signals. The combination of attention-driven feature extraction and diffusion-based denoising provides a complementary mechanism for capturing both informative temporal patterns and noise-resilient representations. These features are provided to grayordinate-specific regressor models, producing an initial prediction of the activation map. In stage 2, these preliminary predictions are enhanced further using a PCA-conditioned Conditional Generative Adversarial Network (PCA-CGAN)^18^. Specifically, PCA restricts adversarial refinement to a low-rank, biologically meaningful subspace, enabling stable learning, reduced overfitting, and spatially coherent task activation maps, while the generator with the proposed activation fidelity loss (AFL) suppresses noise and enhances spatial fidelity, thereby improving prediction performance. This cascaded design—temporal attention-guided diffusion modeling followed by PCA-conditioned adversarial refinement—enables the proposed TADM-CGAN framework to capture both long-range temporal structure and high-resolution spatial organization simultaneously. To the best of our knowledge, this is the first framework that combines temporal attention, diffusion, and CA-conditioned adversarial refinement for grayordinate-specific modeling, establishing a novel, spatially faithful paradigm for generating task-evoked activation maps from rs-fMRI. The key contributions of this work are as follows:

1. Proposed a novel multi-head temporal attention–guided diffusion-based model for the activation map prediction that captures long-range, multiscale temporal dependencies and produces compact, informative latent representations of intrinsic temporal dynamics, eliminating the need for heuristic functional connectivity features.
2. Developed 59,412 optimized grayordinate-specific prediction models using the unique TADM-based embeddings to generate preliminary task activation maps with improved temporal sensitivity and localized prediction accuracy.
3. Designed a novel PCA-conditioned CGAN that refines the initial activation maps using the proposed activation fidelity loss $\mathscr {L}_{AFL}$, incorporating correlation-based and $\ell _1$ reconstruction losses, to generate spatially coherent and noise-suppressed activation maps.
4. Presented the first unified rs-to-task activation map prediction framework that uniquely integrates temporal attention, diffusion-based grayordinate-wise sequence modeling, and PCA-conditioned spatial adversarial refinement, for simultaneous modeling of long-range temporal structure and high-resolution spatial organization, yielding state-of-the-art predictions.

The remainder of this paper is organized as follows: Section “Methods” begins with an overview of the dataset and its preprocessing, followed by a detailed description of the proposed task activation map prediction models. Section "Results and discussion" presents a thorough performance analysis accompanied by an ablation study. Section "Conclusion and future scope" concludes the paper with possible directions for future work.

## Methods

### Dataset and preprocessing

The study presented herein utilized the Human Connectome Project (HCP) S900 new release dataset, comprising rs-fMRI and corresponding task activation maps from 336 healthy adults. All the subjects were preprocessed using the HCP minimal pipeline^19^, which consists of head motion correction, normalization, and projection into grayordinate space. As the cortical region is known to be primarily associated with activation maps^6,20^, the present analysis focused on the 360 cortical parcels obtained using the HCP multimodal parcellation (MMP). The cortical parcels consist of 59412 grayordinates, each with a total of 1200 time points, resulting in a *59412× 1200* dimensional input per subject.

With this input, the prediction targets were chosen as the subject-specific HCP task activation maps for seven cognitive domains: Emotion, Language, Working Memory, Motor, Relational, Social, and Gambling. Specifically, from each of these seven domains, one contrast with corresponding contrast-of-parameter estimates (COPEs) is randomly selected to encompass emotional, cognitive, perceptual, and executive functions, offering a diverse set of high-quality spatial targets for evaluating the proposed TADM-CGAN framework. The selected contrasts are:

- **Emotion (COPE1):** Faces > Shapes comparison, highlighting responses in the amygdala and ventral visual pathways.
- **Gambling (COPE2):** Reward > Punishment contrast, involving striatal and orbitofrontal reward networks.
- **Language (COPE3):** Story > Math contrast, targeting left temporal and inferior frontal language regions.
- **Motor (COPE1):** Average movement contrast, capturing somatomotor and cerebellar activations.
- **Relational (COPE2):** Relational > Match contrast, engaging frontoparietal reasoning and control networks.
- **Social (COPE1):** Theory-of-Mind > Random contrast, activating medial prefrontal and temporoparietal areas.
- **Working Memory (COPE9):** 2-back > 0-back contrast, reflecting dorsolateral prefrontal and parietal engagement.

Here, each COPE measures the magnitude of task-related BOLD activity for a specific contrast through a general linear model (GLM)-based *z*-score, and is represented in standard CIFTI grayordinate space with $91{,}282$ vertices. These activation maps, particularly the cortical activation maps with 59412 grayordinates, highlighting the activated regions of the brain cortex during the given task, serve as the targets for our rs-to-task activation map prediction framework. So, for each subject and each grayordinate, the input is modeled as a 1200-length rs-fMRI time series, and the target output of a predictor is set as the corresponding *z*-score value in the task activation map. All the experimental evaluations in this study employed a 5-fold cross-validation strategy, in which the dataset is partitioned into five non-overlapping folds. In each iteration, four folds are used for training, and one fold is held out for testing. Within the training set, a further split was performed, allocating 20% of the data as a validation set for model selection and early stopping, wherever required. This essentially ensured a strict separation of training, validation, and test data to avoid any data leakage. With this background, the next subsection provides a detailed description of the proposed two-stage TADM–CGAN framework, designed to model these grayordinate-wise prediction functions.

**Fig. 1:**
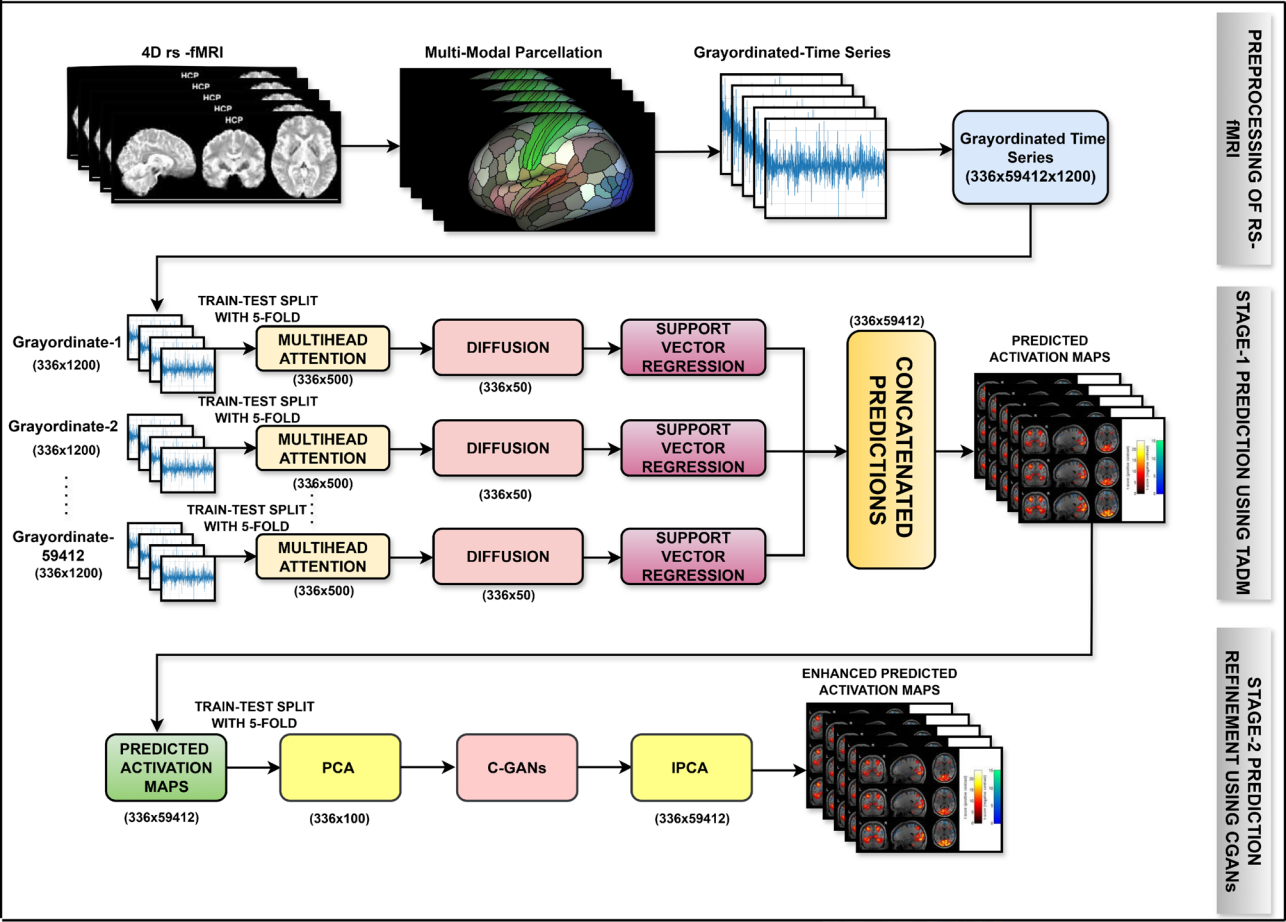
Pipeline of the proposed two-stage TADM–CGAN activation map prediction model.

### Proposed activation map prediction model

**Fig. 2:**
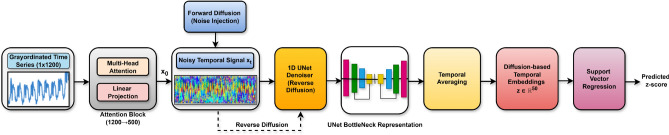
Pipeline of the proposed TADM activation map prediction model.

The proposed two-stage TADM–CGAN activation map prediction framework, as shown in Fig. 1, is divided into two stages, where stage 1, detailed in Fig. 2, generates the initial predictions using the grayordinate-specific temporal attention-guided diffusion models (TADM), while stage 2, detailed in Fig. 4, refines the predictions further using a novel PCA-conditioned CGAN. Each of these prediction stages in the proposed model is explained in detail below.

#### Stage 1: Task activation map prediction using temporal attention–diffusion model (TADM)

**Multi-head Self-Attention for Temporal Modeling**

As explained in the previous subsection, preprocessing of the rs-fMRI data yields a 1200-length time series for each grayordinate, which serves as input to a predictor for the corresponding z-score prediction, thus making this a univariate time series regression problem. However, as fMRI data is often noisy, instead of using it directly, a multi-head self-attention was applied. Eight attention heads operating in parallel enabled the model to capture diverse long-range dependencies across the 1200 rs-fMRI time points, extracting more meaningful temporal information from the original rs-fMRI time series^21,22^. Essentially, it computes interactions across all temporal positions via query $\textbf{Q}$, key $\textbf{K}$, and value $\textbf{V}$ mappings as:

1 $$\begin{aligned} \text {Attention}(\textbf{Q}, \textbf{K}, \textbf{V}) = \text {softmax}\left( \frac{\textbf{Q}\textbf{K}^\top }{\sqrt{d_k}}\right) \textbf{V}, \end{aligned}$$

where $d_k$ is the key dimension. This scaled dot-product attention mechanism evaluates pairwise similarities between queries and keys to derive attention weights, which are further normalized using a softmax operation. These weights determine the relative contribution of each value vector to the output representation, enabling the model to selectively focus on the most relevant contextual information while maintaining numerical stability through scaling by $\sqrt{d_k}$. Finally, a linear projection layer is applied along the temporal dimension to map the input sequence from 1200 to 500 time points, enabling dimensionality reduction while preserving salient temporal information. As revealed by the empirical evaluation, reducing the time series length to 500 enabled multi-head self-attention to selectively emphasize informative temporal patterns in the original rs-fMRI time series while suppressing irrelevant fluctuations, thereby allowing more meaningful and robust feature extraction in the subsequent analysis.

**Diffusion-based latent feature extraction** As stated above, applying the multi-head self-attention temporal attention to the original 1200-length rs-fMRI results in a highly informative 500-attended sequence, better suited for further processing. Diffusion models, with their iterative denoising mechanism, naturally capture both short- and long-range temporal dependencies across multiple scales, thus posing themselves as strong alternatives for extracting the rs-fMRI time series features. So, to obtain the *z*-score prediction for a grayordinate, the corresponding attended rs-fMRI sequence is further modeled using a denoising diffusion framework, which extracts compact, physiologically meaningful latent representations, as explained below.

**Forward diffusion process.** Given an attended time series $\textbf{x}_0 \in \mathbb {R}^{500}$, the forward diffusion process gradually corrupts the signal with Gaussian noise over *T=200* steps. At time step *t*, the noisy sample $\textbf{x}_t$ is drawn as:

2 $$\begin{aligned} \textbf{x}_t = \sqrt{\hat{\alpha }_t}\, \textbf{x}_0 + \sqrt{1-\hat{\alpha }_t}\, \boldsymbol{\epsilon }, \qquad \boldsymbol{\epsilon } \sim \mathscr {N}(\textbf{0}, \textbf{I}), \end{aligned}$$

where $\{\alpha _t\}_{t=1}^T$ is a predefined noise schedule and

3 $$\begin{aligned} \hat{\alpha }_t = \prod _{s=1}^{t} \alpha _s \end{aligned}$$

is the cumulative product governing the proportion of the original signal preserved at step *t*. In particular, a linear variance schedule is followed, in which the noise variance $\beta _t$ is increased linearly from $1 \times 10^{-4}$ to 0.02 over *T=200* timesteps. The corresponding $\alpha _t = 1 - \beta _t$ defines the signal retention at each step, and the cumulative product $\hat{\alpha }_t = \prod _{s=1}^{t} \alpha _s$ controls the progressive corruption of the input signal. At each training iteration, a timestep *t* is sampled uniformly from a discrete distribution over $\{1, \dots , T\}$, and Gaussian noise is added to the input according to this schedule.

**Reverse denoising model.** The denoiser is implemented as a 1D UNet composed of residual convolutional blocks:

4 $$\begin{aligned} \textbf{h}_{\ell +1} = \sigma \!\left( g_{\ell }(\textbf{h}_{\ell }) + s_{\ell }(\textbf{h}_{\ell }) \right) , \end{aligned}$$

where *ℓ* denotes the depth of the U-Net, $\textbf{h}_{\ell }$ is a UNet feature map, $g_\ell$ denotes the two-layer convolutional path, $s_\ell$ is a *1 × 1* shortcut projection, and *σ* is ReLU. The encoder of the UNet consists of three sequential residual blocks that progressively increase the channel dimensionality from 1 to 64, 128, and 256 channels, respectively. Each residual block contains two 1D convolutional layers with kernel size 3, followed by group normalization (8 groups) and ReLU activation, along with a skip connection implemented via identity mapping or a *1 × 1* convolution when dimensions differ. The final UNet feature map, obtained from the output of the last residual block, serves as the input to the noise prediction head. Hierarchical feature abstraction is achieved through progressive channel expansion in the encoder, while the bottleneck block produces the 50-length latent representation:

5 $$\begin{aligned} \textbf{z} = \text {mean}_{t}\!\left( \text {UNet}_{\text {bottleneck}}(\textbf{x}_t) \right) \in \mathbb {R}^{50}. \end{aligned}$$

**Fig. 3:**
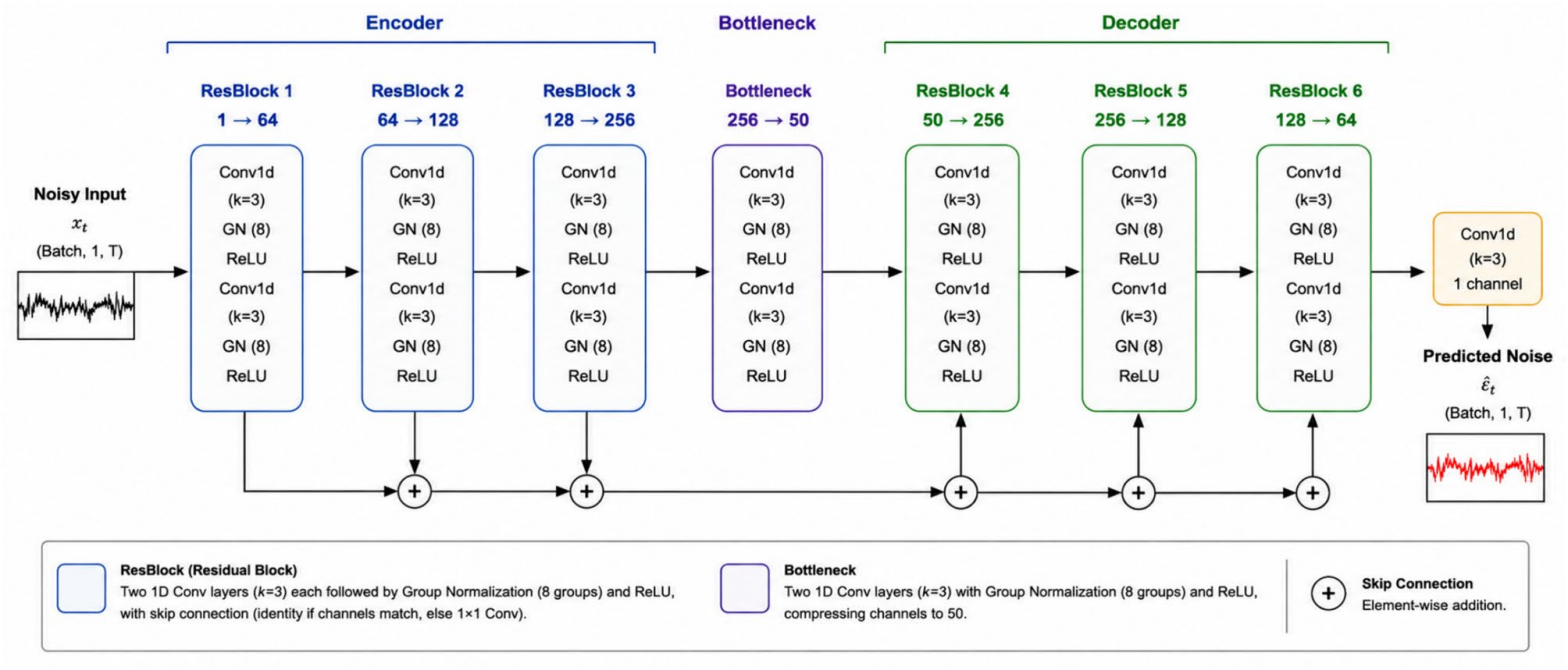
Architecture of the proposed 1D residual U-Net denoising network.

The length of the latent representation was set to 50, as this yielded the optimal prediction performance. The decoder mirrors the encoder structure with three residual blocks that progressively reduce the channel dimensions from 50 to 256, 128, and 64, respectively. Skip connections between encoder and decoder stages are implemented using element-wise addition to preserve temporal information. A final convolutional layer maps the decoded features back to a single-channel output for noise prediction, as depicted in Fig. 3. Notably, temporal resolution is preserved throughout the network, and hierarchical feature abstraction is achieved via channel expansion rather than explicit temporal downsampling.

After training, the denoising UNet model $f_\theta$ receives $\textbf{x}_t$ and timestep *t* and outputs the noise estimate $\hat{\boldsymbol{\epsilon }}_\theta$:

6 $$\begin{aligned} \hat{\boldsymbol{\epsilon }}_\theta = f_\theta (\textbf{x}_t, t). \end{aligned}$$

The training objective follows the standard distributive diffusion probabilistic model (DDPM) noise-prediction loss:

7 $$\begin{aligned} \mathscr {L}_{\text {DDPM}} = \mathbb {E}_{\textbf{x}_0, t, \boldsymbol{\epsilon }} \left[ \left\| \boldsymbol{\epsilon } - f_\theta (\textbf{x}_t, t) \right\| _2^2 \right] . \end{aligned}$$

**Latent Representation Extraction.** After training, each attended time series is encoded by passing it through the UNet and extracting the bottleneck activation. Temporal averaging yields a 50-length latent vector per grayordinate:

8 $$\begin{aligned} \textbf{z}_{i,j} \in \mathbb {R}^{50}, \end{aligned}$$

where *i* indexes subjects and *j* indexes grayordinates. Stacking all representations yields the embedding:

9 $$\begin{aligned} \textbf{Z} \in \mathbb {R}^{N_{\text {sub}} \times N_{\text {gray}} \times 50}. \end{aligned}$$

where, $N_{\text {sub}}$ denotes number of subjects and $N_{\text {gray}}$ denotes number of grayordiantes. These diffusion-derived embeddings capture nonlinear temporal structure, multi-scale dynamics, and intrinsic variability of the BOLD sequence, forming the input to the grayordinate-wise regressors in the subsequent stage.

##### Grayordinate-wise regression for z-score prediction

As explained above, the trained TADM yields a matrix of shape $(336\times 59412\times 50)$, where for each of the 336 subjects, each of the 59412 grayordinates is assigned a 50-dimensional temporal descriptor. These temporal descriptors are used as predictors of the regressors, where one unique regressor is trained per grayordinate to map these descriptors to their corresponding task activation value (COPE *z*-scores). Specifically, 59412 SVR regressors were trained, as they provided superior predictions when compared to the other standard regressors. The detailed comparative analysis is included in the ablation study in section Ablation study. For each SVR regressor, a radial basis function (RBF) kernel was employed to capture nonlinear relationships, and performance was assessed using 5-fold cross-validation. Since tuning all 59412 models is computationally expensive, the SVR with default hyperparameters model was used, which also achieved strong and consistent performance across all task contrasts, as demonstrated in the experimental results. These SVR-predicted activation maps, obtained using the algorithm 1, form the coarse stage 1 estimates, which are subsequently refined by the PCA-conditioned conditional GAN (CGAN) in stage 2, as explained next.

**Algorithm 1:**
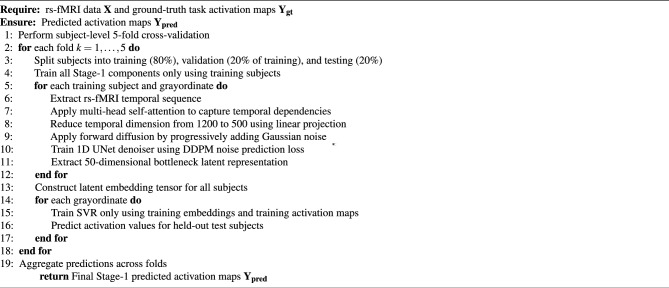
Stage-1: TADM-Based Activation Map Prediction.

#### Stage2: task activation map refinement using PCA-based conditional generative adversarial network

As stated earlier, the second stage of our proposed framework refines the task activation maps predicted by stage 1 using a PCA-driven GAN. Given the predicted activation maps $\textbf{Y}_{\text {pred}}$ obtained from stage 1, the objective is to learn a refinement function

10 $$\begin{aligned} f:\ \textbf{Y}_{\text {pred}} \rightarrow \textbf{Y}_{\text {ref}}, \end{aligned}$$

generating refined predictions $\textbf{Y}_{\text {ref}}$. Specifically, for 268 training subjects and 59412 grayordinates, $\textbf{Y}_{\text {ref}},\ \textbf{Y}_{\text {pred}} \in \mathbb {R}^{268 \times 59412}$. However, direct learning in the original space $\mathbb {R}^{268 \times 59412}$ results in a curse of dimensionality as

11 $$\begin{aligned} 59412 \gg 268 \;\; \rightarrow \;\; \operatorname {rank}(\textbf{Y}) \le 268, \end{aligned}$$

making adversarial training in $\mathbb {R}^{59412}$ highly under-constrained and ill-posed. But with strong spatial correlations and network-level organization, the activation maps typically exhibit low intrinsic dimensionality^23,24^ and hence can be reasonably approximated in a reduced-dimensional subspace. So, instead of learning refinement in original $\mathbb {R}^{59412}$, a conditional GAN architecture *G* is defined as:

12 $$\begin{aligned} G:\ \mathbb {R}^{100} \rightarrow \mathbb {R}^{100}, \end{aligned}$$

operating exclusively in the PCA-derived low-rank subspace $\mathbb {R}^{100}$ with 100 principal components, as the same leads to optimal predictions with the limited available training data. Particularly, the generator *G* is implemented using a series of fully connected layers with layer normalization and LeakyReLU activations, which iteratively refines PCA-derived task features while being conditioned on the subject-specific embeddings. The generator was optimized using the proposed activation fidelity loss (AFL):

$$\begin{aligned} \mathscr {L}_{AFL} = \mathscr {L}_{\text {adv}} + \lambda _1 \mathscr {L}_{\ell _1} + \lambda _2 \mathscr {L}_{\text {corr}} + \lambda _3 \mathscr {L}_{\text {cos}}, \end{aligned}$$

where $\mathscr {L}_{\text {adv}}$ promotes realism in the generated features, $\mathscr {L}_{\ell _1}$ enforces grayordinate-level reconstruction accuracy, $\mathscr {L}_{\text {corr}}$ enhances correlation among predicted and actual activation structures, and $\mathscr {L}_{\text {cos}}$ maintains angular consistency within the PCA subspace, resulting in the enhanced predictions. Following the empirical evaluation, the weighting factors were set to $\lambda _1 = 20$, $\lambda _2 = 20$, and $\lambda _3 = 5$. To ensure stable training, *G* was first pretrained for 10 epochs using only the $\ell _1$ reconstruction and correlation losses, providing a reliable initialization before introducing the adversarial component. The discriminator *D* was then trained for a maximum of 100 epochs (with early stopping based on validation correlation) to distinguish ground-truth PCA features from those produced by *G*, encouraging the generator to produce outputs that are both realistic and consistent with the underlying task structure. Both the generator and the discriminator were trained using the Adam optimizer with a learning rate of $1 \times 10^{-4}$, and a mini-batch size of 16. To improve stability, Gaussian noise with small variance was added to the input embeddings during training. So, during training, PCA embeddings of the first-stage-predicted activation maps $\textbf{W}_{\text {pred}}$, are provided to the generator *G*, which produces the PCA embeddings $\hat{\textbf{W}}_{\text {ref}}$. Subsequently, the discriminator updates the generated $\hat{\textbf{W}}_{\text {ref}}$ using PCA embeddings of the ground-truth activation maps $\textbf{W}_{\text {gt}}$ and the AFL loss, to finally obtain refined PCA embeddings $\textbf{W}_{\text {ref}}$.

**Fig. 4:**
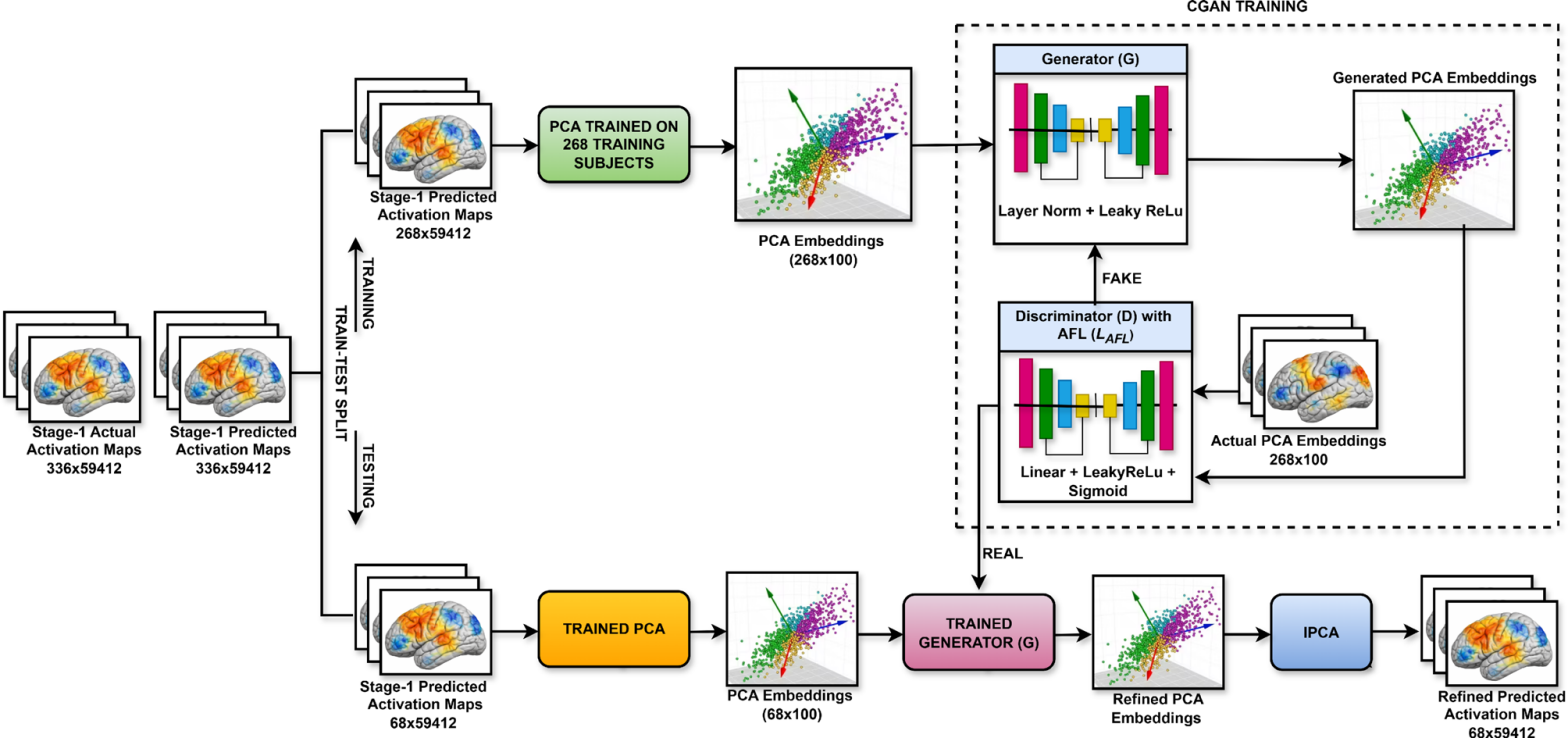
Pipeline of the proposed PCA-CGAN architecture for enhancing task activation map predictions.

Finally, during inference, the refined PCA embeddings for the test subjects are obtained as,

13 $$\begin{aligned} \textbf{W}_{\text {ref}} = G\!(\textbf{W}_{\text {pred}}) \end{aligned}$$

by applying the trained *G* to the PCA embeddings of their first-stage prediction, $\textbf{W}_{\text {pred}}$. Importantly, during inference, the trained generator *G* takes only the PCA embedding of the stage-1 predicted activation maps as input and produces refined PCA embeddings, without using ground-truth embeddings as conditioning inputs. The refined PCA embeddings $\textbf{W}_{\text {ref}}$ are then projected back onto the cortical surface using the inverse PCA transform to obtain the desired activation maps $\textbf{Y}_{\text {ref}}$, as detailed in Fig. 4 and algorithm 2. As expected, on the held-out test set, the GAN-refined outputs exhibited a significantly higher correlation and lower reconstruction error compared to the baseline diffusion model outputs, which can be attributed to two key factors. First, incorporating correlation-based loss $\mathscr {L}_{\text {corr}}$ and $\ell _1$ reconstruction loss into $\mathscr {L}_{\text {AFL}}$ directly links the training process to neuroscientific evaluation metrics, resulting in activation patterns that more closely match the ground-truth. Second, conditioning the GAN on PCA features constrains adversarial refinement to the biologically meaningful low-dimensional subspace, thereby enforcing implicit regularization and spatial coherence, leading to stable learning with limited neuroimaging samples.

**Algorithm 2:**
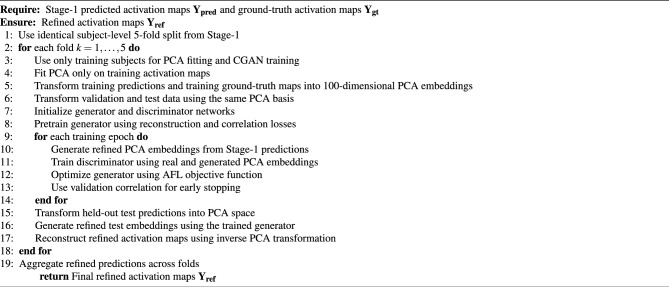
Stage-2: PCA-CGAN Refinement.

## Results and discussion

This section begins with a brief description of prediction performance metrics used in this work, followed by the detailed performance analysis of our proposed models. Further, it compares these results with those of existing state-of-the-art methods.

### Quantitative performance evaluation

To quantitatively assess the accuracy of the predicted task activation maps against the ground-truth task fMRI activations, three complementary evaluation metrics: the Pearson correlation coefficient (r), the Dice similarity coefficient (DSC), and the Area Under the ROC Curve (AUC) were employed in this work, each capturing a distinct characteristic of model performance, as detailed below.

1. Pearson correlation coefficient (r): It measures the linear relationship between predicted and actual activation intensities across all grayordinates for a given subject and is defined as:

14 $$\begin{aligned} r = \frac{ \sum _{i=1}^{N} \left( y_i - \bar{y}\right) \left( \hat{y}_i - \overline{\hat{y}}\right) }{ \sqrt{\sum _{i=1}^{N} \left( y_i - \bar{y}\right) ^2} \sqrt{\sum _{i=1}^{N} \left( \hat{y}_i - \overline{\hat{y}}\right) ^2} } \end{aligned}$$

where $y_i$ and $\hat{y}_i$ denote the true and predicted activation values at grayordinate *i*, respectively, and *N* is the total number of grayordinates. Ranging between −1 and 1, a higher *r* value indicates better alignment between the predicted and ground-truth activations.

2. Dice similarity coefficient (DSC): It measures the spatial overlap between the predicted and actual activation regions after thresholding and is given by:

15 $$\begin{aligned} \textrm{Dice} = \frac{ 2 \left| A \cap B \right| }{ \left| A \right| + \left| B \right| } \end{aligned}$$

where *A* and *B* denote the sets of activated grayordinates in the predicted and true maps, respectively. It ranges from 0 to 1, with 0 indicating no overlap and 1 indicating the perfect overlap. Although the prediction model predicts continuous activation values, neuroscientific interpretations typically rely on thresholded activation maps, thus making the dice coefficient a biologically meaningful measure of how well the predicted activation areas align with true activations^5,12^.

3. Area under the ROC curve (AUC): It evaluates the discriminative power of the model in distinguishing between active and inactive grayordinates across all thresholds. It is computed as the area under the receiver operating characteristic (ROC) curve, defined by:

16 $$\begin{aligned} \textrm{AUC} = \int _{0}^{1} \textrm{TPR}(\textrm{FPR}) \, d(\textrm{FPR}) \end{aligned}$$

where TPR and FPR denote the true positive and false positive rates, respectively. Similar to DSC, it ranges between 0 and 1, with a higher value indicating better performance.

To ensure a rigorous and reproducible evaluation, a 5-fold cross-validation strategy was employed throughout the study. The dataset was partitioned into five non-overlapping folds, where in each iteration, four folds (80%) were used for training, and one fold (20%) was held out for testing. Within the training set, a further split was performed, allocating 20% of the data as a validation set for model selection and early stopping, wherever required. All model components, including the temporal attention module, diffusion-based feature extractor, PCA transformation, CGAN, and regression models, were trained exclusively using the training data within each fold to prevent data leakage. The validation set was used to monitor performance and tune training dynamics such as early stopping, while the test set remained completely unseen during training. During inference, the trained models were applied to the held-out test subjects to generate final predictions. All preprocessing and transformations are recomputed independently within each fold to ensure strict separation between training and evaluation data. Subsequently, the performance metrics described above were used to assess the predictive performance of all models on rs-fMRI data from the HCP dataset, as described in section Dataset and preprocessing.

Starting with the predictions obtained using TADM discussed in section Stage 1: Task activation map prediction using temporal attention–diffusion model (TADM), table 1 summarizes the evaluation metrics when regressed with SVR. As can be observed therein, with impressive correlations ranging between 0.6006 to 0.7305, the TADM model demonstrates consistent performance across all seven contrasts. The DICE score also remains moderate to high for all the tasks, suggesting a good spatial overlap between the actual and predicted task activation maps. Furthermore, the AUC values exceed 0.79 for all task contrasts, demonstrating their reliability in distinguishing between active and inactive brain regions. Overall, these findings indicate that the proposed TADM provides robust task activation map predictions, which subsequently serve as a strong baseline for CGAN refinement.

After validating the performance of the TADM model, a similar performance analysis was conducted for the proposed TADM-CGAN model, which augments the TADM model with CGAN, and the corresponding results are summarized in table 2. Comparing these results obtained by incorporating the CGAN-based refinement stage with the earlier results in table 1, clearly indicates the consistent and significant improvement in the prediction correlations from 0.7305, 0.6957, 0.6605, 0.6286, 0.6006, 0.6486, 0.6983 to 0.8513, 0.8264, 0.7984, 0.7944, 0.7762, 0.7969, 0.8379 for relational, social, language, motor, emotion, gambling, and working memory contrasts, respectively. Thus, the introduction of the CGAN-enabled refinement stage results in an appreciable average gain of 0.15 in correlation across different contrasts, highlighting the advantage of the proposed two-stage prediction framework, further corroborating the proposed hypothesis. Interestingly, the prediction results also reveal that even the poorest prediction, which occurs for the emotion task, has a remarkable correlation of 0.776, thus attesting to the robustness of the proposed framework to various tasks. After assessing the correlations, a similar analysis was extended to evaluate AUC and DC metrics, the results of which are summarized in table 1 and 2. Consistent with the correlation results, the inclusion of the CGAN-based refinement stage led to substantial improvements in both AUC and DC, with average improvements of 0.05 and 0.06, respectively, across contrasts, reiterating the importance of the proposed two-stage prediction framework.

**Table 1:** Mean ± standard deviation of *r*, DICE, and AUC scores across 5-fold cross-validation using the TADM model.

S.No	Contrast	r	DICE	AUC
1	RELATIONAL	*0.7305 ± 0.005*	*0.728 ± 0.005*	*0.904 ± 0.003*
2	SOCIAL	*0.696 ± 0.001*	*0.728 ± 0.007*	*0.892 ± 0.002*
3	LANGUAGE	*0.653 ± 0.010*	*0.652 ± 0.007*	*0.792 ± 0.006*
4	MOTOR	*0.629 ± 0.004*	*0.812 ± 0.007*	*0.853 ± 0.003*
5	EMOTION	*0.601 ± 0.013*	*0.572 ± 0.009*	*0.881 ± 0.009*
6	GAMBLING	*0.649 ± 0.010*	*0.690 ± 0.004*	*0.861 ± 0.006*
7	WM	*0.698 ± 0.007*	*0.717 ± 0.006*	*0.886 ± 0.004*

**Table 2:** Mean ± standard deviation of *r*, DICE, and AUC scores across 5-fold cross-validation using the TADM-CGAN model.

S.No	Contrast	r	DICE	AUC
1	RELATIONAL	*0.851 ± 0.006*	*0.784 ± 0.003*	*0.957 ± 0.002*
2	SOCIAL	*0.826 ± 0.007*	*0.784 ± 0.006*	*0.949 ± 0.001*
3	LANGUAGE	*0.798 ± 0.008*	*0.728 ± 0.006*	*0.876 ± 0.006*
4	MOTOR	*0.795 ± 0.004*	*0.893 ± 0.006*	*0.928 ± 0.002*
5	EMOTION	*0.776 ± 0.005*	*0.702 ± 0.005*	*0.961 ± 0.005*
6	GAMBLING	*0.797 ± 0.009*	*0.726 ± 0.008*	*0.929 ± 0.004*
7	WM	*0.838 ± 0.007*	*0.755 ± 0.003*	*0.949 ± 0.004*

**Table 3:** Quantitative evaluation of different threshold settings across seven tasks using Dice coefficient and AUC metrics. Here, T refers to the threshold value.

S.No	Task	T = 40%		T = 45%		T = 55%		T = 60%	
		Dice	AUC	Dice	AUC	Dice	AUC	Dice	AUC
1	RELATION	0.8361	0.8825	0.8268	0.8907	0.8157	0.9092	0.8099	0.9188
2	SOCIAL	0.8117	0.8550	0.8028	0.8647	0.7921	0.8883	0.7900	0.9016
3	LANGUAGE	0.8097	0.8516	0.7927	0.8549	0.7657	0.8671	0.7557	0.8756
4	MOTOR	0.8517	0.8945	0.8332	0.8948	0.7922	0.8951	0.7699	0.8953
5	EMOTION	0.7699	0.7981	0.7497	0.8039	0.7162	0.8201	0.7037	0.8309
6	GAMBLING	0.8260	0.8654	0.8128	0.8718	0.7903	0.8868	0.7797	0.8950
7	WORKING	0.8374	0.8828	0.8254	0.8892	0.8060	0.9039	0.7989	0.9120

Apart from the average performance measures, the results in tables 1 & 2 also indicate the standard deviation (SD) in the metrics, obtained across different folds. Remarkably, both TADM and TADM-CGAN exhibit a low average SD of 0.006 and 0.007, respectively, in correlation, indicating their compatibility with different splits. A similar trend is observed for DICE and AUC, wherein the TADM achieves average SD of 0.005 and 0.006, respectively, while the TADM-CGAN predictions result in average SD of 0.003 and 0.004, respectively, highlighting the model’s strong generalization capability and its suitability for reliable task activation prediction across diverse subject subsets. Although inspired by previous studies^5,12^, the Dice similarity coefficient and AUC metrics hitherto were obtained using a median-thresholding strategy; to further investigate the robustness of the proposed framework with respect to threshold selection, a comparative performance analysis was conducted by varying the threshold levels from 40% to 60%. The corresponding quantitative results across all seven tasks are summarized in table 3. The results demonstrate that the overall performance trends remain consistent across different threshold settings, indicating that the proposed framework is not overly sensitive to a specific threshold choice.

**Fig. 5:**
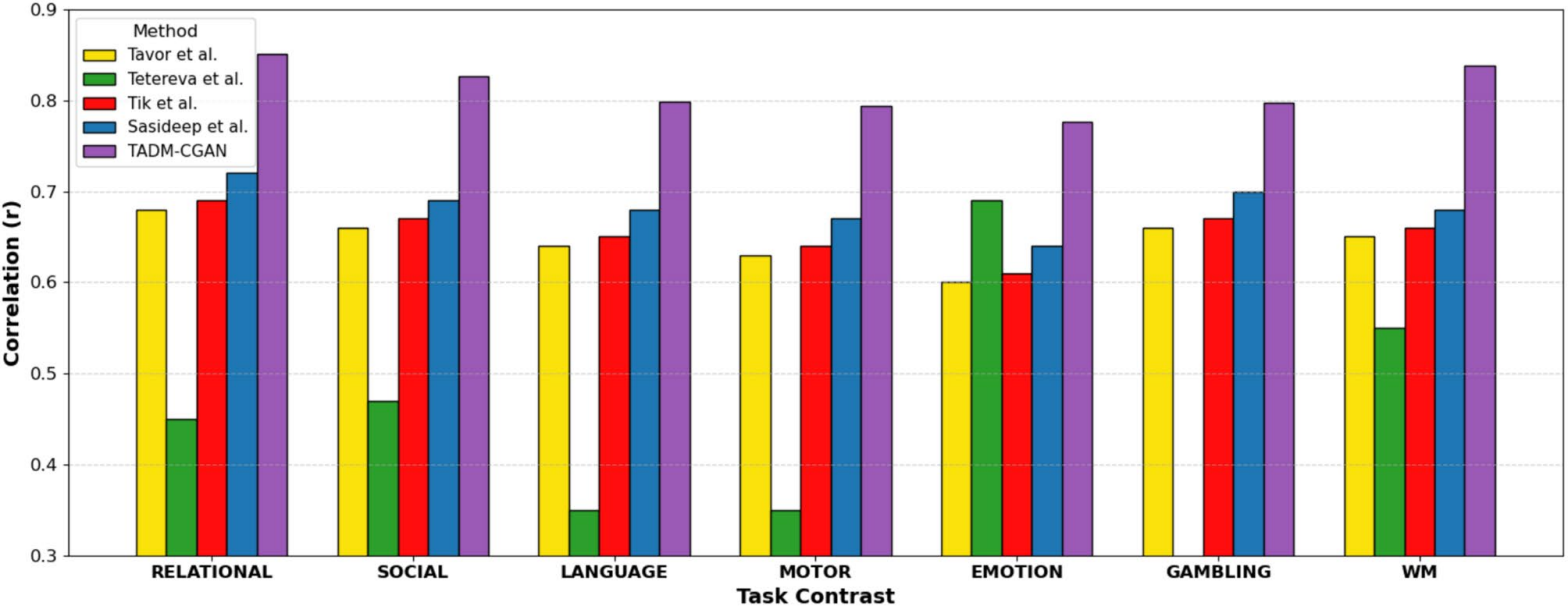
Performance comparison of different activation map prediction methods across different task contrasts (Tavor et al.^5^, Tetereva et al.^7^, Tik et al.^9^, Sasideep et al.^15^).

After a thorough performance evaluation of the proposed models, Fig. 5 compares their prediction performance with that of existing state-of-the-art methods. As evident therein, the proposed TADM-CGAN model exhibits superior performance compared to all existing methods, as well as the proposed TADM model, across all evaluation metrics and task contrasts. Specifically, compared to the best existing method^5,15^, the proposed TADM-CGAN achieves a substantial average improvement of 0.165 in correlation across different task contrasts, thereby advocating its practical utility for rs-to-task activation map prediction. This significant improvement in prediction performance can be attributed to the proposed novel prediction framework, which uniquely integrates temporal attention-guided diffusion-based grayordinate-wise sequence modeling with PCA-conditioned GAN refinement, facilitating the simultaneous modeling of long-range temporal structure and high-resolution spatial organization. Following the comprehensive quantitative performance assessment, the next subsection presents representative visualizations of predicted versus actual task activation maps to facilitate a better understanding of the results.

### Visual assessment of predicted activation maps

To facilitate the visual assessment of the prediction results, the predicted activation maps obtained from the proposed TADM-CGAN model are mapped onto $fs_{LR}32k$ surface^19^ using CIFTI grayordinate space with 59412 grayordinates.

**Fig. 6:**
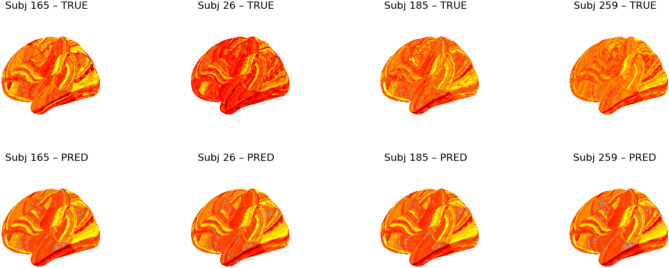
Predicted vs actual activation maps for Relational task contrast.

**Fig. 7:**
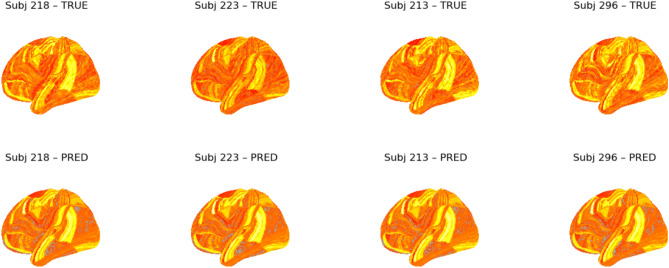
Predicted vs actual activation maps for Social task contrast.

**Fig. 8:**
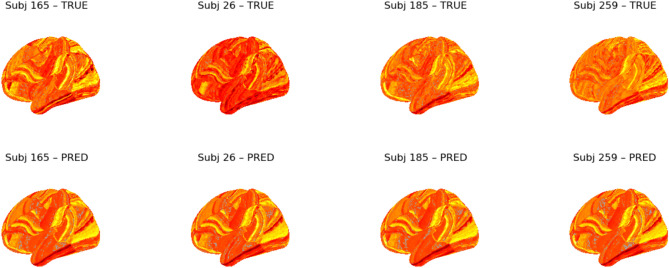
Predicted vs actual activation maps for Language task contrast.

**Fig. 9:**
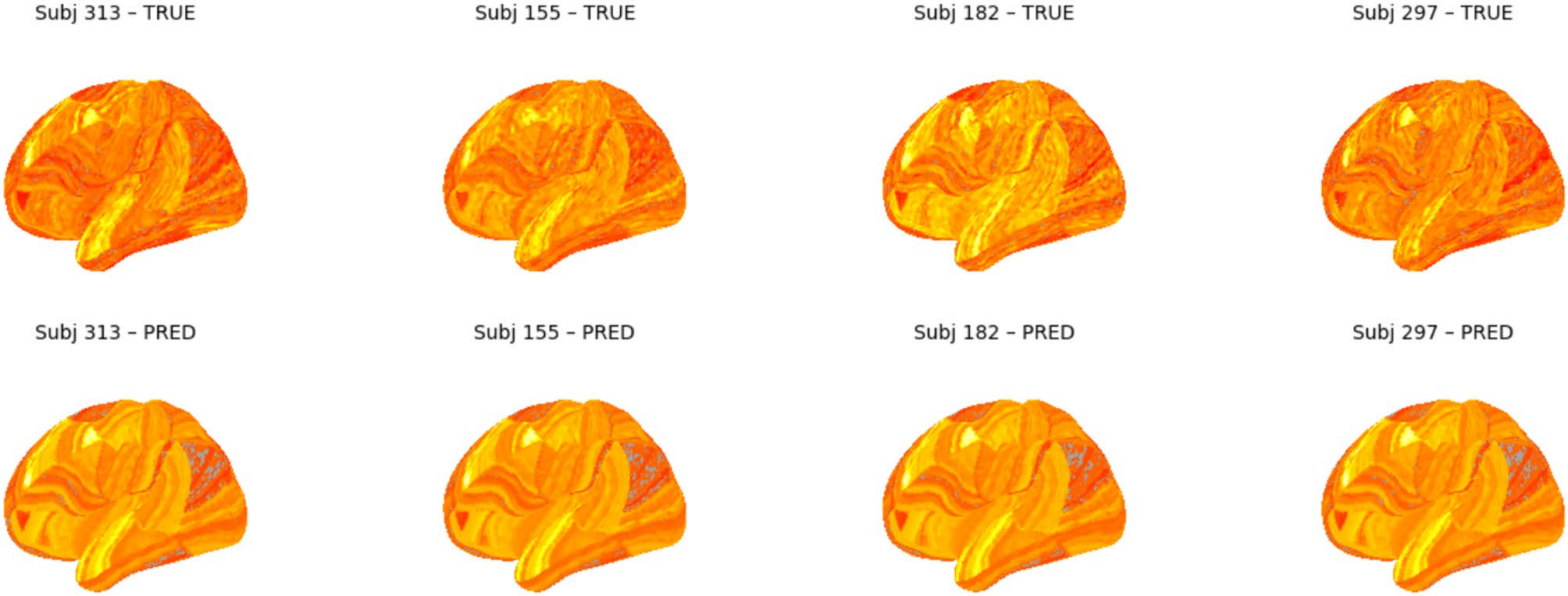
Predicted vs actual activation maps for Motor task contrast.

**Fig. 10:**
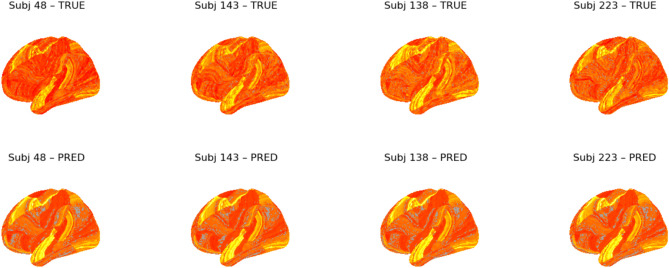
Predicted vs actual activation maps for Emotion task contrast.

**Fig. 11:**
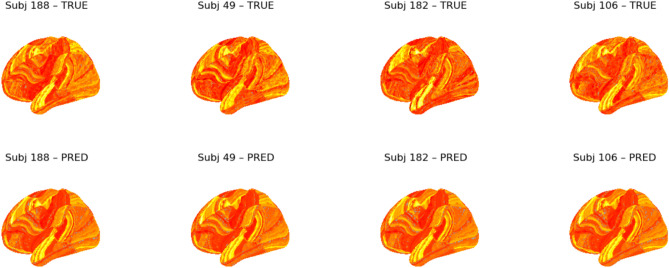
Predicted vs actual activation maps for Gambling task contrast.

**Fig. 12:**
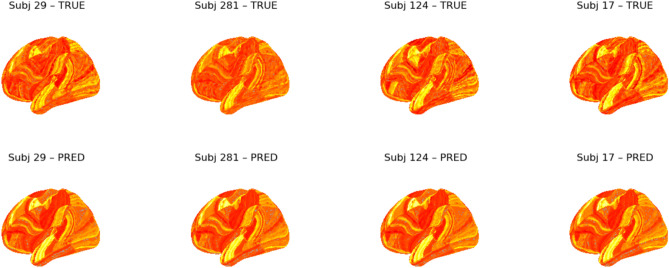
Predicted vs actual activation maps for Working Memory task contrast.

Although predictions were obtained for all test subjects, for representation purposes, four random subjects were selected for each task contrast. Figures 6, 7, 8, 9, 10, 11, and 12 demonstrate the predicted vs. actual activation maps for these subjects, where the above row corresponds to the actual activations while the bottom one represents the predicted activations.

Overall, the visualizations confirm that the significant correlation values observed in the quantitative evaluation correspond to meaningful spatial overlap as well.

Delving deeper into the observed predictions, Fig. 6, corresponding to the relational task, shows more overlap in the lateral prefrontal cortex and parietal association areas, known for their activation in relational tasks. For social contrast, Fig. 7 indicates a strong correspondence between actual vs predicted in the temporo-parietal junction and the medial prefrontal cortex, typically known for social cognition and TOM (theory-of-mind).

In Fig. 8, the predicted language task activation maps strongly overlap with the actual maps in canonical language-related regions, namely Broca’s and Wernicke’s areas, which are the primary components of the language task-network.

Figure 9, representing the motor task activations, shows a strong spatial correspondence in the primary cortex and the primary sensory cortex, the essential components of the motor network, indicating the model’s efficacy in capturing the intrinsic functional characteristics of the brain. In line with the previous observations, Fig. 10, corresponding to the emotion task, indicates a strong overlap between the actual and predicted task activation maps in the key regions of the emotion-activated network, namely the anterior cingulate cortex, and ventromedial prefrontal cortex visual regions.

**Fig. 13:**
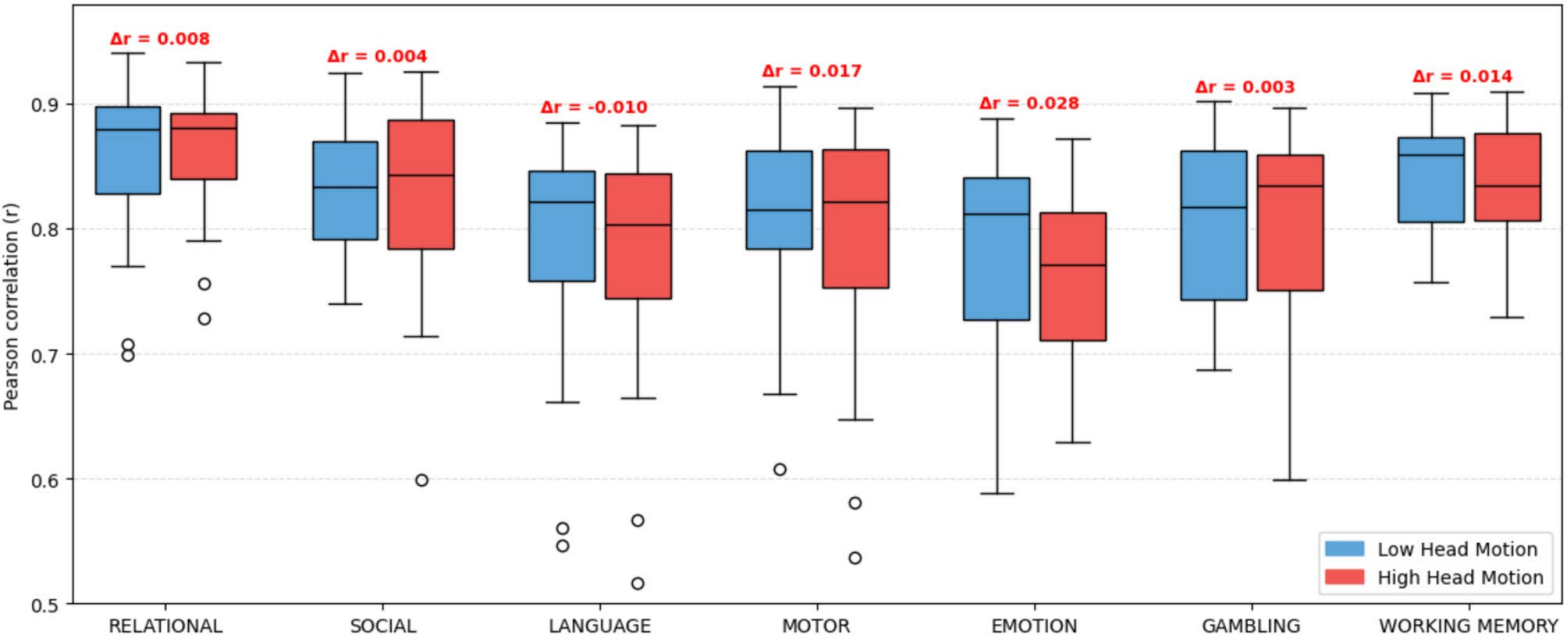
Effect of head motion error on prediction performance of TADM-CGAN.

Figure 11, representing the gambling task, also shows a strong agreement between the actual and predicted in the medial prefrontal cortex, and orbitofrontal cortex, the regions primarily involved in decision making and reward processing. Finally, for a working memory task, visualized in Fig. 12, a significant overlap is observed between the predicted and actual activation maps, particularly in the dorsolateral prefrontal cortex, the posterior parietal cortex, and the anterior cingulate cortex, the key regions of the working-memory activation pattern.

Altogether, these insights from the visual assessments demonstrate a strong spatial correspondence between the predicted and actual activation maps, particularly in well-established task-induced brain regions, thereby further attesting to the TADM-CGAN’s spatial fidelity. Having verified the prediction efficacy of the proposed model, both quantitatively and qualitatively, the next subsections analyze its robustness to noisy and out-of-sample data, to further validate its practical utility.

### Noise-robustness and cross-dataset generalization of TADM-CGAN model

#### Noise-robustness analysis

To evaluate the robustness of the proposed prediction model to the noisy data acquisitions, a systematic head motion error analysis was performed. Apart from being a typical source of noise, head motion also acts as a natural proxy for noisy data acquisitions, particularly expected from the target non-compliant populations. To this end, following Power et. al^25^., the mean frame displacement (FD), quantifying the subject motion, was obtained for every test subject using their motion regressors. Specifically, mean FD was calculated as the sum of absolute frame-to-frame differences across the six rigid-body motion parameters. Based on the median of FD, the test subjects were categorized into two equal-sized groups, viz., low head motion (LHM) group and high head motion (HHM) group. Following this, task activation predictive performance was evaluated for both groups to observe the group-wise performance differences across all seven task contrasts, as summarized in Fig. 13. As expected, the comparative analysis indicates a slight but consistent drop in prediction accuracy for subjects in the HHM group, with an average decrease of 0.012 in correlation, when compared with the LHM group. This marginal reduction in prediction efficacy for noisy acquisitions essentially demonstrates that the proposed TADM-CGAN can be extended to non-compliant populations, including the elderly, infants, and subjects with disabilities, provided that careful preprocessing, such as motion correction and motion artifact pipelines, is employed.

#### Cross dataset generalization

After verifying the robustness of the proposed TADM-CGAN model to the noisy fMRI acquisitions, to evaluate its generalizability, its performance was further assessed using the rs-fMRI data of 40 subjects from the Chinese Human Connectome Project (CHCP) dataset^26^. Unlike the HCP dataset used hitherto, the CHCP dataset has a different acquisition protocol, scanner hardware, preprocessing strategies, noise profiles, and population characteristics, resulting in 634 time points. So, to evaluate the cross-dataset generalization of the proposed prediction models, both the proposed models, trained using the HCP dataset, were tested on the unseen rs-fMRI data from the CHCP dataset. To this end, the CHCP time series, comprising 634 time points, was used directly as input without temporal resampling. The multi-head attention block in the TADM-CGAN maps variable-length temporal inputs to a fixed-dimensional representation (500 features), followed by diffusion-based embedding extraction, enabling consistent processing across datasets with differing temporal resolutions. Subsequently, the average prediction performance metrics were computed for each model, as tabulated in table 4. Comparison of these results with the results in tables 1 & 2, obtained for the HCP dataset, essentially demonstrates the strong generalizability of the proposed models. Despite significant differences in the datasets, the correlations are increased for a few contrasts, viz., Emotion and Social, while they drop nominally for a few like Relational, Motor, and Working Memory, further advocating its practical applicability. After the thorough performance analysis presented hitherto, the next subsection deals with the associated ablation study.

**Table 4:** Prediction performance of the proposed models on the CHCP dataset.

S.No	Contrasts	TADM			TADM+CGAN		
		r	DICE	AUC	r	DICE	AUC
1	RELATIONAL	0.6755	0.6734	0.8487	0.7963	0.7285	0.9019
2	SOCIAL	0.7107	0.7434	0.9072	0.8414	0.7986	0.9635
3	LANGUAGE	0.6055	0.5970	0.7436	0.7434	0.6728	0.8280
4	MOTOR	0.5736	0.7570	0.7976	0.7394	0.8385	0.9388
5	EMOTION	0.6156	0.5875	0.8960	0.7912	0.7169	0.9763
6	GAMBLING	0.5936	0.6351	0.8056	0.7419	0.6715	0.8731
7	WM	0.6433	0.6622	0.8311	0.7829	0.7005	0.8940

### Ablation study

**Fig. 14:**
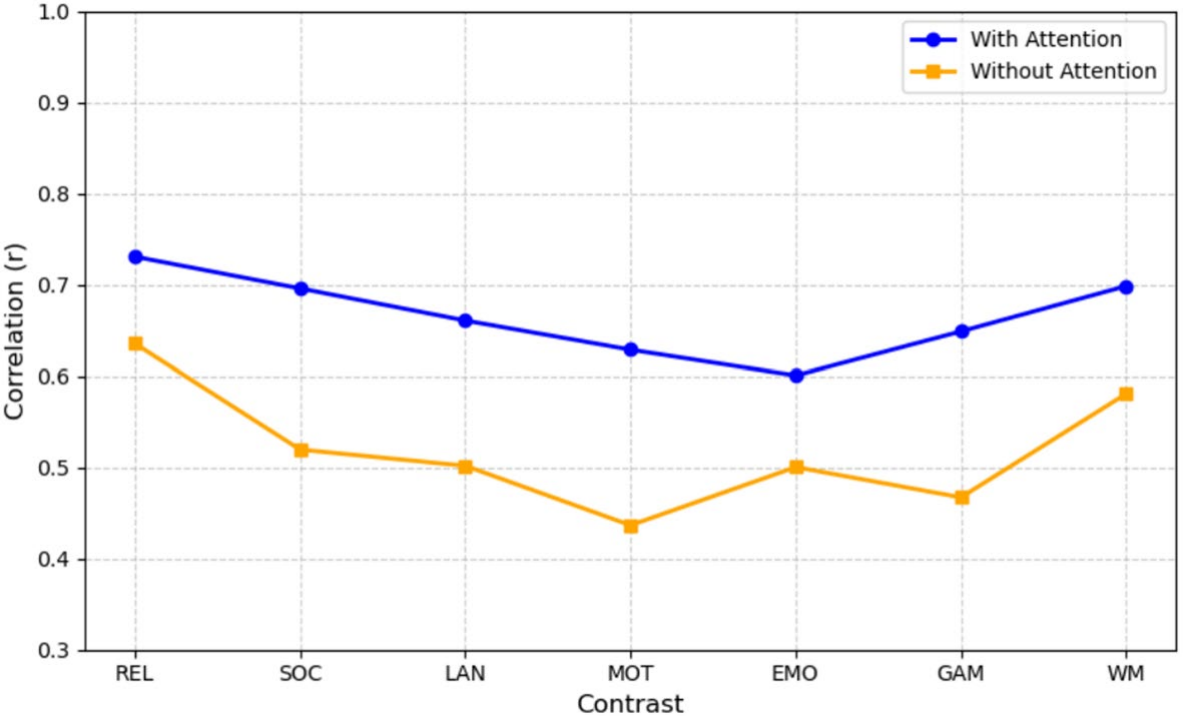
Prediction performance of TADM with and without attention for different contrasts.

This subsection details the ablation study performed herein for optimizing the proposed prediction models, which involved analyzing the effect of the attention module, PCA, generator loss function, and different regressors in the proposed TADM-CGAN framework. Starting with the first stage, i.e., TADM, to analyze the effect of the attention module on overall prediction performance, an ablation study was conducted, examining the model’s performance with and without attention. The comparative results, summarized in Fig. 14, demonstrate that the introduction of attention results in a consistent and significant improvement in prediction correlations, with an average improvement of 0.16, across all tasks. This necessarily corroborates our idea of employing the attention module in conjunction with the diffusion model for the effective temporal feature extraction from the rs-fMRI time series.

**Fig. 15:**
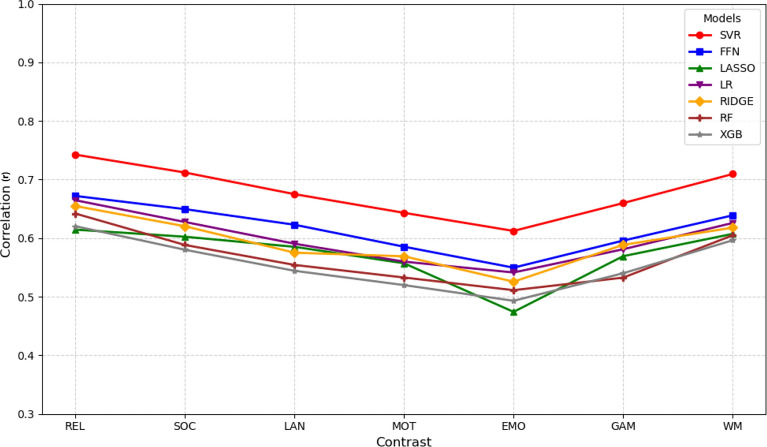
Prediction performance of TADM using different regression models for different contrasts.

**Fig. 16:**
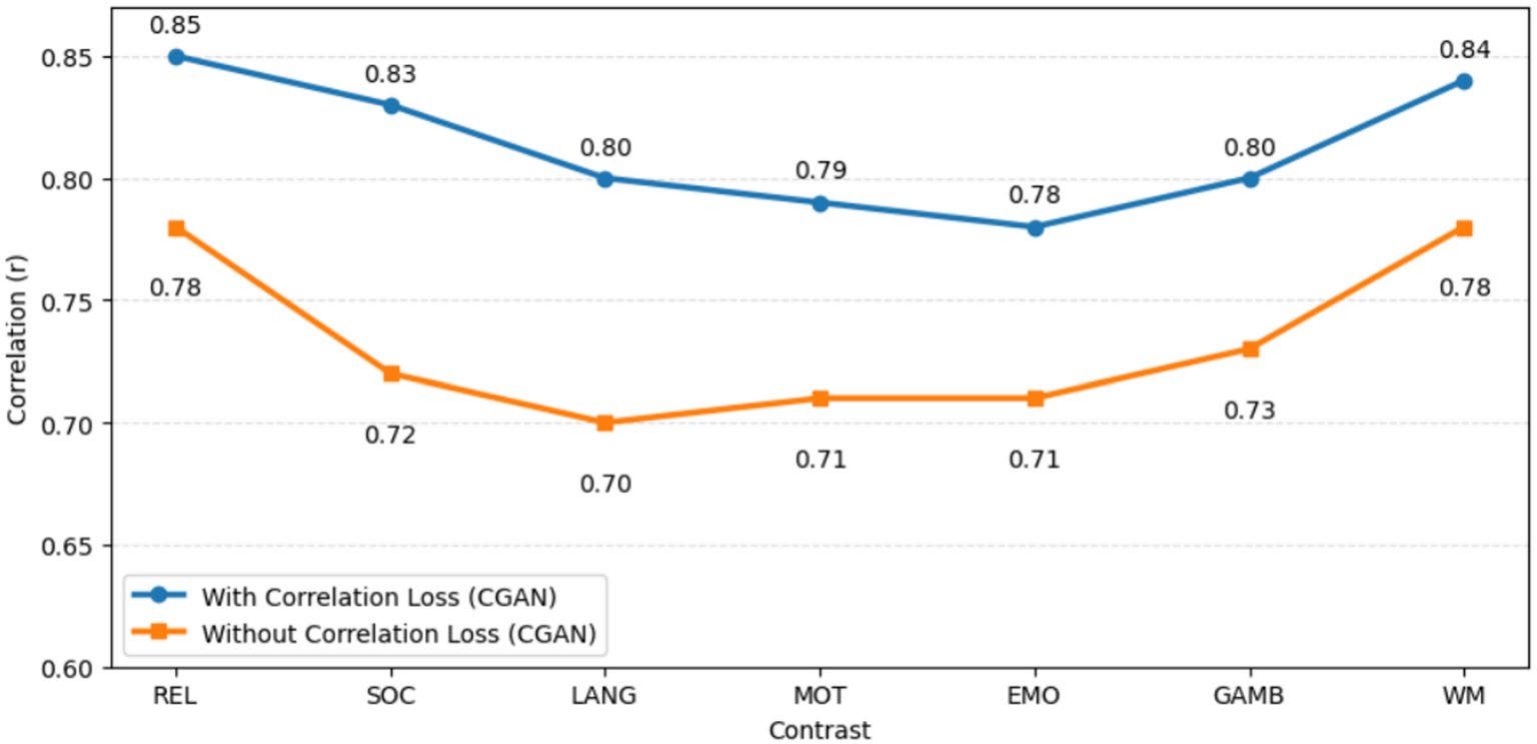
Prediction performance of TADM-CGAN with and without correlation loss for different contrasts.

**Fig. 17:**
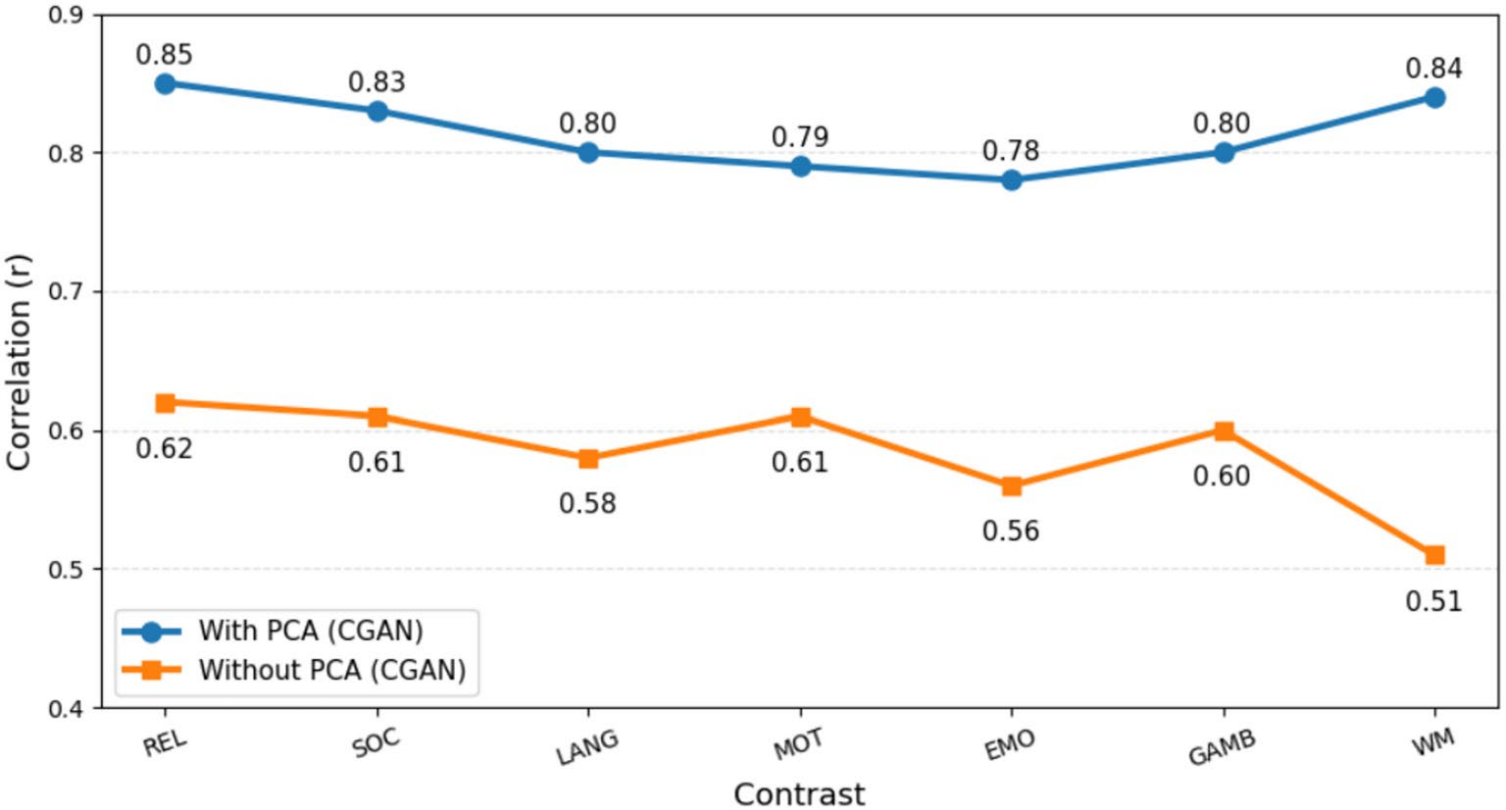
Prediction performance of TADM-CGAN with and without PCA for different contrasts.

Apart from feature extraction, the choice of regressor also affects the regression performance, in general. Hence, to determine the optimal regressor in the proposed TADM architecture, an ablation analysis was conducted to find the regressor yielding the best prediction correlations. Specifically, the prediction performance of TADM was examined by employing different standard regressors, including SVR, feed-forward network, LASSO regressor, linear regressor, ridge regressor, random forest regressor, and XGBoost regressor. As summarized in Fig.15, among all these regressors, SVR outperformed the others, yielding consistently higher correlations across all tasks with a mean improvement of 0.11, and hence was used as a regressor in all further analysis.

After optimizing the first stage of the proposed framework, a further ablation study was conducted to examine the efficacy of the proposed generator loss function $\mathscr {L}_{AFL}$ in the second stage involving CGAN. To this end, the predictive performance of the CGAN model was evaluated, with and without the proposed correlation loss term, as compared in Fig. 16. As can be observed therein, the inclusion of the correlation loss term in the proposed generator loss function remarkably boosts prediction performance, resulting in an average gain of 0.08 in correlation, validating its utility in improving prediction fidelity. Additionally, the significance of dimensionality reduction was also investigated by comparing the performance of the proposed model with and without PCA, as illustrated in Fig. 17. As clearly highlighted in the comparison, with an average rise of 0.22 in correlations across all tasks, the inclusion of PCA substantially improves the prediction performance, strongly corroborating the advantage of working in the lower-dimensional dominant subspace. Overall, these ablation results highlight the importance of multi-head attention and SVR in the stage-1 TADM architecture, and the significance of the proposed $\mathscr {L}_{AFL}$ and PCA in the stage-2 CGAN architecture, for the optimal task activation map prediction framework. Although the above correlation-based ablation analysis helps finalize the design choices for the proposed model, their effects are further analyzed using statistical significance analysis presented below.

#### Statistical significance

**Table 5:** Statistical comparison between TADM-CGAN and TADM across task contrasts.

Task	Model	Baseline	p-value (t/W)	Cohen’s *d*
Relational	0.8513	0.6380	<0.001/<0.001	3.09
Social	0.8264	0.5865	<0.001/<0.001	3.35
Language	0.7984	0.5324	<0.001/<0.001	3.16
Motor	0.7944	0.5089	<0.001/<0.001	3.43
Emotion	0.7762	0.4823	<0.001/<0.001	3.28
Gambling	0.7969	0.5285	<0.001/<0.001	2.97
Working Memory	0.8379	0.5957	<0.001/<0.001	3.54

**Table 6:** Statistical comparison between TADM with attention and TADM without attention across task contrasts.

Task	With Attention	Without Attention	p-value (t/W)	Cohen’s *d*
Relational	0.7305	0.6253	*<0.001*/*<0.001*	2.32
Social	0.6960	0.6118	*<0.001*/*<0.001*	2.06
Language	0.6530	0.5898	*<0.001*/*<0.001*	2.12
Motor	0.6290	0.6100	*<0.001*/*<0.001*	2.98
Emotion	0.6010	0.5635	*<0.001*/*<0.001*	2.33
Gambling	0.6490	0.6001	*<0.001*/*<0.001*	2.56
Working Memory	0.6980	0.5110	*<0.001*/*<0.001*	2.00

**Table 7:** Statistical comparison between TADM-CGAN with PCA and TADM-CGAN without PCA across task contrasts.

Task	With PCA	Without PCA	p-value (t/W)	Cohen’s *d*
Relational	0.8513	0.6253	*<0.001*/*<0.001*	3.45
Social	0.8264	0.6118	*<0.001*/*<0.001*	3.01
Language	0.7984	0.5898	*<0.001*/*<0.001*	3.53
Motor	0.7944	0.6100	*<0.001*/*<0.001*	3.50
Emotion	0.7762	0.5635	*<0.001*/*<0.001*	3.30
Gambling	0.7969	0.6001	*<0.001*/*<0.001*	3.10
Working Memory	0.8379	0.5110	*<0.001*/*<0.001*	3.60

**Table 8:** Statistical comparison between TADM-CGAN with AFL and TADM-CGAN without AFL across task contrasts.

Task	With AFL	Without AFL	p-value (t/W)	Cohen’s *d*
Relational	0.8513	0.7813	*<0.001*/*<0.001*	2.15
Social	0.8264	0.7197	*<0.001*/*<0.001*	2.23
Language	0.7984	0.6944	*<0.001*/*<0.001*	2.44
Motor	0.7944	0.7096	*<0.001*/*<0.001*	2.00
Emotion	0.7762	0.7110	*<0.001*/*<0.001*	2.10
Gambling	0.7969	0.7251	*<0.001*/*<0.001*	1.98
Working Memory	0.8379	0.7701	*<0.001*/*<0.001*	2.00

To isolate the contribution of each major component in the proposed TADM-CGAN framework, a statistical significance analysis was performed. Specifically, paired t-tests, Wilcoxon signed-rank tests, and Cohen’s *d* effect size analyses were conducted for the following comparisons: i) proposed TADM-CGAN versus only TADM, ii) TADM with and without the temporal attention module, iii) TADM-CGAN with and without PCA conditioning, and iv) TADM-CGAN with and without the AFL objective. The detailed statistical analyses across all task contrasts are summarized in Tables 5–8. In all cases, the proposed configurations achieved statistically significant improvements (*p<0.001*) with large effect sizes, further validating the effectiveness of the proposed framework and its individual components. Overall, the statistical analyses consistently demonstrate that each component of the proposed framework contributes positively to the final prediction performance, with the complete TADM-CGAN architecture achieving the most substantial and statistically significant improvements across all task contrasts. Following the thorough empirical evaluation presented so far, the next subsection discusses the computational complexity and implementation details of the proposed model, which are helpful for its practical deployment and replication.

### Computational complexity and implementation details

All experiments were conducted on an Intel Xeon Platinum 8352Y CPU (2.20 GHz, dual processor), 256 GB RAM, and an NVIDIA RTX A6000 GPU with 48 GB VRAM. The complete training of the proposed TADM-CGAN pipeline, including temporal attention, diffusion-based feature extraction, PCA transformation, CGAN refinement, and grayordinate-wise SVR models, requires approximately 40 minutes per contrast, whereas the inference time is approximately 5 minutes per contrast.

The 59,412 SVR models are trained in parallel across CPU cores, making the overall computation tractable. Using a compact 50-dimensional latent representation significantly reduces the computational burden of regression, enabling efficient training despite the large number of models. The proposed framework can be executed on a single high-end GPU with sufficient RAM, making it feasible for typical neuroimaging setups. While computational cost scales linearly with the number of grayordinates and subjects, this can be mitigated through parallelization or region-wise aggregation strategies. The pipeline’s modular design further enables flexible deployment and scalability. To ensure that the proposed model can be independently reproduced by the research community, a public GitHub repository containing the full implementation of the proposed framework has been created and can be accessed via the link^27^.

## Conclusion and future scope

This work introduces a novel two-stage TADM-CGAN prediction framework for estimating task activation maps directly from the temporal information of rs-fMRI, representing a clear departure from conventional approaches, primarily relying on functional connectivity–based features. Furthermore, unlike most existing methods, the proposed framework operates at the grayordinate level, wherein the unique TADM features extracted from 59,412 individual grayordinates are modeled using their respective predictors, forming the first stage of the proposed framework. Subsequently, a CGAN–based refinement stage is employed using the proposed generator loss function $\mathscr {L}_{AFL}$ and a PCA-based dimensionality reduction, substantially improving the initial predictions. State-of-the-art prediction performance of the resulting cascaded framework across different datasets and signal qualities, and its superiority over the existing methods attests to its practical applicability in rest-to-task activation map prediction.

Although the presented TADM-CGAN framework yielded state-of-the-art prediction performance, outperforming the existing methods, the framework could be extended from the current univariate time series modeling to a multivariate time-series formulation, which may better capture inter-grayordinate dependencies. Further, incorporation of graph-signal modeling in the prediction framework can potentially improve the predictions by enabling richer modeling of spatio-temporal interactions inherent in rs-fMRI data.

## Data Availability

The data used in this study are available from HCP and CHCP.

## Code availability

All the codes used in this study are made publicly available in a GitHub repository^27^.
